# Beneficial Role of Vitexin in Parkinson’s Disease

**DOI:** 10.21315/mjms2023.30.2.2

**Published:** 2022-04-18

**Authors:** Musa Mustapha, Che Norma Mat Taib

**Affiliations:** 1 Department of Human Anatomy, Faculty of Medicine and Health Sciences, Universiti Putra Malaysia, Selangor, Malaysia; 2 Department of Human Anatomy, Faculty of Basic Medical Sciences, College of Medical Sciences, Ahmadu Bello University, Zaria, Nigeria

**Keywords:** vitexin, Parkinson’s disease, nuclear factor erythroid 2-related factor 2, nuclear factor-kB, MAO-B, phosphatidyl inositol-3 kinase, Akt

## Abstract

Today, Parkinson’s disease (PD) is the foremost neurological disorder all across the globe. In the quest for a novel therapeutic agent for PD with a multimodal mechanism of action and relatively better safety profile, natural flavonoids are now receiving greater attention as a potential source of neuroprotection. Vitexin have been shown to exhibit diverse biological benefits in various disease conditions, including PD. It exerts its anti-oxidative property in PD patients by either directly scavenging reactive oxygen species (ROS) or by upregulating the expression of nuclear factor erythroid 2-related factor 2 (Nrf2) and enhancing the activities of antioxidant enzymes. Also, vitexin activates the ERK1/1 and phosphatidyl inositol-3 kinase/Akt (PI3K/Akt) pro-survival signalling pathway, which upregulates the release of anti-apoptotic proteins and downregulates the expression of pro-apoptotic proteins. It could be antagonistic to protein misfolding and aggregation. Studies have shown that it can also act as an inhibitor of monoamine oxidase B (MAO-B) enzyme, thereby increasing striatal dopamine levels, and hence, restoring the behavioural deficit in experimental PD models. Such promising pharmacological potential of vitexin could be a game-changer in devising novel therapeutic strategies against PD. This review discusses the chemistry, properties, sources, bioavailability and safety profile of vitexin. The possible molecular mechanisms underlying the neuroprotective action of vitexin in the pathogenesis of PD alongside its therapeutic potential is also discussed.

## Introduction

Parkinson’s disease (PD) is a progressive and irremediable neurodegenerative movement disorder primarily incident in older population (1). The Global Burden of Disease study of 2016 have reported PD as the leading neurological movement disorder with a high incidence. From 1990 to 2015, over six million people developed PD and it is anticipated that by the year 2040, this number will double (2). Previous studies have shown that factors such as increasing industrialisation, increasing longevity, and decreasing smoking rates could further increase the incidence of PD, leading to increased morbidity and mortality (2). PD negatively affects the quality of life for both patients and their families and adds to significant economic and institutional costs on patients’ families and the society in general (3).

The precise aetiology of PD is still obscure, however, it is believed to be caused by a complex interplay between mitochondrial dysfunction, oxidative stress, apoptotic cell death, cellular protein aggregation and misfolding, neuroinflammation, excitotoxicity, loss of trophic factors and promotion of other cell death pathways leading to a degeneration of dopaminergic nigrostriatal neurons in the brain (4). The core clinical features seen in PD patients include tremor, bradykinesia, rigidity and postural instability (5). With progression of the disease, PD patients also exhibit non-motor symptoms, including dementia, dysautonomia depression, anxiety, somnolence, attention deficit, hyposmia and restless leg syndrome (6). The histopathological hallmarks of PD are dopaminergic neuronal loss in the substantia nigra pars compacta (SNpc), diminished dopamine content in the striatum and accumulation of α-synuclein (SNCA) in the form of Lewy bodies (LB) in the striatum (7). At present, the approved and commonly used anti-PD drugs, such as L-DOPA (a dopamine precursor) and selegiline or rasagiline (a monoamine oxidase inhibitor), only provide symptomatic relief (2) and have been linked with adverse effects, such as compulsive and impulsive behaviour, dementia, depression and dyskinesia, after long-term administration (6). Studies have reported that none of the approved anti-PD drugs can effectively treat the disease because of the singleton pathway characteristics they exhibit while attacking the complex PD processes (6). Therefore, any drug that acts on only one pathway or target is unlikely to tone down the composite patho-aetiological cascade that leads to PD. Thus, a drug approach with a multi-modal mechanism of action and high safety profile will provide optimum treatment benefits compared to the current therapeutic strategies (8).

Natural pharmaceutical products are now receiving greater attention as a source of neuroprotective drugs as they can maintain normal cellular interaction in the brain and reduce the loss of neuronal functions in pathological circumstances (9). In the field of PD research, natural plant polyphenolic compounds have now gained considerable interest because of their potential multiple mechanisms of action, such as the inhibition of protein aggregation and misfolding, antioxidative and anti-inflammatory properties, the modulatory effect on apoptosis and cell survival, effects on cell cycle genes as well as signalling pathways and their relatively low toxicity (8). In this context, flavonoids, a class of polyphenols derived from plant products and ubiquitously found in the human diet, act as a promising candidate for anti-parkinsonism (10). Flavonoids are commonly found in vegetables, fruits, seeds, grains, tea, nuts and traditional medicinal herbs (11), and the recommended daily dietary intake of flavonoids is 50 mg–800 mg (1). Till date, more than 6,000 flavonoid types have been isolated from natural sources (12). Generally, flavonoids have low molecular weight with a 15 carbon skeleton and 2 benzene rings linked via a heterocyclic pyran ring (13). Based on the oxidation state and the presence of sugar moiety attached to carbon (C-glycosides) or hydroxyl (O-glycosides) on the basic flavonoid structure (aglycone), flavonoids are classified into neoflavonoids, flavonols, flavanals or catechins, flavanonols, isoflavones, anthrocyanins, flavonones and flavones (13). These groups of flavonoids show diverse biological functions, however, the flavones are the most extensively studied group because of their promising biological properties observed in many in vivo and in vitro studies (14).

The presence of double bond between C2 and C3 flavonoid skeleton and a ketone in position 4 of the ring distinguishes flavones from other flavonoid types (15). The C-glycoside flavones exhibit more resistance and stability to enzymatic, acidic and alkaline hydrolysis compared to the O-glycoside flavones; the diverse biological activities of C-glycoside flavones are attributed to their higher stability (16). Flavones primarily exhibit anti-inflammatory, antioxidative, antiviral and anti-carcinogenic properties, among others, mediated by their interplay with many key enzymes and signalling cascades involving cytokines and regulatory transcription factors (17). Though flavones show promising results in preclinical studies, their bioavailability, toxicological profile and clinical efficacy in human subjects still need to be explored thoroughly before use (18).

The naturally occurring flavones are apigenin, luteolin and chrysin and are commonly found in the fruit skin, red wine, buckwheat, red pepper and tomato skin, which we inadvertently consume in our diet (19). Studies have reported some variations in the daily average intake of flavones across different cohorts, including 1.35 mg/d in American women (20), 3.05 mg/d in Chinese female adolescents (21) and 4.85 mg/d in European adults (22). The apigenin flavone is the most remarkably explored natural bioactive flavone-type molecule by researches because of its promising therapeutic functions (23). The primary constituents of apigenin flavones are glycosylated apigenin, vitexin, apiin, isovitexin and rhoifolin (24). Vitexin, c-glycosylated apigenin is now gaining considerable attention from researchers as a result of its multi-modal mechanisms of action and diverse biological advantages in different disease conditions, including neurodegenerative diseases (25). Extensive literature search has revealed that most of the reviews discussed the neuroprotective effects of general flavonoids in neurodegenerative diseases (26). However, a recent review by Angelopoulou et al. (1) specifically discussed the neuroprotective potential of chrysin, a flavone, in PD patients. To the best of our knowledge, no review has focused specifically on the potential neuroprotective properties of vitexin in PD patients. Even though this area is still in its nascent stage, the few available studies of vitexin in PD models have shown a glimmer of hope for PD patients.

Therefore, to fill the lacuna in knowledge, this review discusses the chemistry, properties, sources, bioavailability and safety profile of vitexin to provide a reference for future preclinical and clinical PD studies. The possible mechanism of action and therapeutic potential of this important nutraceutical agent in PD pathogenesis is also elucidated to promote its clinical application. The literature used in this review was collected from credible scientific databases, such as ScienceDirect, Scopus, PubMed and Google Scholar, using the relevant search keywords, including Parkinson’s disease, vitexin, neuroprotection, flavonoids and flavones.

### Chemistry and Properties of Vitexin

The scientific name of vitexin is 5,7-dihydroxy-2-(4-hydroxyphenyl)-8-{(2S,3R,4R, 5S,6R)-3,4,5-trihydroxy-6-(hydroxymethyl) oxan-2-yl} chromen-4-one). It is also known with other names, including 4,5,7-trihdroxyflavone, 8-C-glucosylapegenin, apigenin 8-c-glucoside and orientoside (Chemspider.com). Some of the derivatives of vitexin are isovitexin, vitexin-2-O-rhamnoside (VOR), rhamnopyranosyl-vitexin, vitexin-2-O-xyloside (VOX) and methylvitexin (isoembigenin) (27). Its empirical formula is C_21_H_20_O_10_ and the molecular weight is 432.38 g/mol.

**Figure 1:**
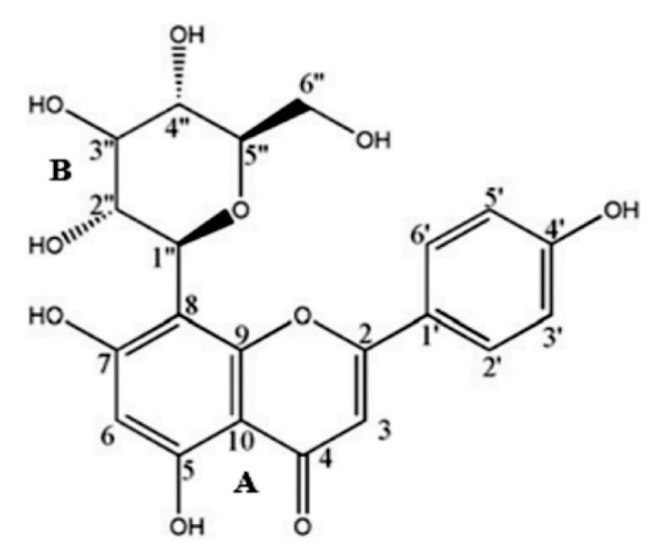
Chemical structure of vitexin

As shown in the chemical structure below (Figure 1), vitexin contains an apigenin moiety: the 5,7-dihydroxyl group at A-ring, the 4’-hydroxyl group at B-ring, and the 2,3-double bond in conjugation with a 4-oxo group. The chemical composition on vitexin structure was confirmed previously by Guimarães et al. (28) through carbon-nuclear magnetic resonance (C-NMR) and hydrogen-nuclear magnetic resonance (H-NMR) analyses of *Serjania erecta* leaves with the aid of a Bruker DXP-300 spectrometer that operates at 125 MHz and 500 MHz, respectively. The seven hydroxyl (OH) groups present in vitexin are said to account for its strong biological effects. Studies have shown that it is the O-di-hydroxyl structure present in the vitexin ring that contributes to its radical scavenging potential (29). The C-8 glucoside present in vitexin is responsible for the decrease in its bond dissociation enthalpy, thus adding to its radical scavenging property (27). The stable radical order in vitexin has been reported as 4’-OH>7-OH N>5-OH, which is responsible for its potent antioxidant property (30). Vitexin has a planar structure with C-O-C bond angle as 120.9°, C-O bond length as 1.376 Å and the dihedral angle as 179.2° (31). Vitexin occurs as a yellow crystalline solid in its pure form with greater than 95% purity.

Yu et al. (32) isolated 2.1 mg vitexin from 100 mg of ethyl acetate extract of *Trollius chinensis* Bunge with a 96% purity using high-speed counter-current chromatography. In a similar study conducted by Xue et al. (33), vitexin with 99% purity was obtained from the *Crataegus pinnatifida* (*C. pinnatifida*) leaves using high-performance liquid chromatography (HPLC). In aqueous extract and commercial products of Andean *Passiflora* species, Sepúlveda et al. (34) also obtained vitexin with 99% purity using HPLC. Similarly, in another study, 0.5 g vitexin was obtained from the leaves of *C*. *pinnatifida. var major* with a purity of greater than 99% using HPLC, and its structure was fully characterised using H-NMR and C-NMR (35). Vitexin has a poor water solubility (7.6172 μg/mL), which limits its dissolution, and thus, accounts for its poor bioavailability (36). To increase its solubility, vitexin is dissolved in organic solvents, such as dimethyl sulfoxide (DMSO) and dimethylformamide (37). Carrier molecules, including carbon nano powder (36), self-micro emulsifying delivery system (38), Solutol HS 15 and polymeric micelles of Pluronic P123 (39), have been previously used to increase the efficacy of vitexin. In aqueous buffers, vitexin is sparingly soluble, and to achieve maximum solubility, it should be dissolved in DMSO first before diluting with a buffer of choice: 0.5 mg/mL vitexin in a 1:1 solution of DMSO: phosphate buffered saline (PBS) at pH of 7.2.

Vitexin is unstable at room temperature and should be stored at −20 °C. For use in the experiments, it is recommended that aqueous vitexin solution should not be stored for more than one day (37). In terms of stability in analytical solution, Raghu and Agrawal (40), concluded that vitexin standard and sample preparation is stable for up to 24 h with percentage difference as 0.8% and 1.8%, respectively, at room temperature (25 °C). The ultraviolet-visible (UV) spectroscopy value of vitexin is UV *γ*_max_ nm: 215, 270 and 332. Yu et al. (32) obtained UV (MeOH) *γ*_max_ at 235 nm, 269 nm and 335 nm as the spectrophotometric value of vitexin extracted from *Trollius chinensis* Bunge using a high-speed counter-current chromatography. The maximum spectral wavelength of vitexin isolated from leaves extracts of *Justicia gendarussa* plant was 335 nm (40).

### Vitexin: Sources, Bioavailability and Safety Profile

Vitexin is found in the leaves, fruits, flowers and barks of many medicinal plant species, such as *Ficus deltoidea* (41), *Trollius chenensis* (42); *Spirodela polyrhiza* (43), *Acer palmatum* (44), *Mung bean* (45), hawthorn (46), buckwheat (47), *Parkinsonian aculeate* (48) and *Passiflora incarnate* (49). A study has shown that hawthorn leaves provides the richest source of vitexin (29). The concentration of vitexin in some of the species of hawthorn has been reported by previous studies as follows:

- *C. cuneate* leaves: 0.1905 mg/g–0.5616 mg/g (50)
- *C. huphenesis* fruit: 0.33 mg/g–1.08 mg/g (50)
- *C. microphylla* leaves: 0.034 mg/g (51)
- *C. pinnatifida* fruits and leaves: 0.028 mg/g–1.30 mg/g and 0.22 mg/g–9.53 mg/g, respectively (52)
- *C. sanguinea* fruits 0.38 mg/g (51)

Since vitexin is ubiquitous in many dietary plants that we consume inadvertently, the estimation of its daily intake is important to elucidate its precise correlation with health outcomes. Some of the previous reports on daily dietary intake of flavones were directed specifically on apigenin. For example, Lefort and Blay (53) reported that the estimated daily consumption of apigenin in humans is between 0 mg and 18 mg. The average apigenin intake is 3 ± 1 mg/day for European cohort (54), 4.23 mg/day for Chinese cohort (55), 0.13 mg/day–1.35 mg/day for American middle-aged and elderly women (56) and 0.45 mg/day for Australian adults (57). However, there is a dearth of documented reports addressing explicitly recommended dietary intake of vitexin (37). There is a strong need to research the daily intake of vitexin so that its biological health effects can fully be appreciated.

The therapeutic potential of vitexin like any natural product is dependent on important factors, like bioavailability and probable in vivo cellular and target tissue concentrations. According to Peng et al. (18), poor absorption, broad metabolism and speedy clearance of vitexin and its metabolite in the gut is the cause of its poor bioavailability. In another study by Zu et al. (36), vitexin was rapidly eliminated from the blood as a result of poor solubility associated with impaired drug bioavailability (36). In another study by Liang et al. (58), vitexin rhamnoside was eliminated with 1.07 ± 0.26 L/h/kg as the systemic clearance, 0.72 ± 0.15 h as the half-life, 1.09 ± 0.22 L/kg as the volume of distribution and 0.92 ± 0.14 h as the time to maximum plasma concentration following intravenous administration, leading to its low bioavailability. Xue et al. (33) obtained 94%, 30% and 5%, as the intestinal, gastric and hepatic first-pass effects, respectively, leading to the low bioavailability of vitexin. However, studies have suggested that the use of liposomes, nanoparticles and micelles as drug delivery systems could enhance vitexin bioavailability in in vitro (59). Thilakarathna and Rupasinghe (60) suggested that another way of increasing the bioavailability of flavonoid is via food supplements.

Generally, flavonoids have not been linked with any adverse health effect and are generally considered to be beneficial (37). The apigenin flavone is generally known for their low toxicity (24). Furthermore, Choo et al. (41) observed no signs of vitexin toxicity at the highest dose of 2 g/kg administered orally to normoglycaemic mice and induced diabetic rats. In an in vitro study of *F. deltoidea* methanol extract, which is believed to contain high levels of vitexin, no sign of toxicity was observed at a single oral gavage dose of 6,400 mg/kg or daily dose of 200 mg/kg for 4 weeks (61). Rosa et al. (62), observed that after incubating RAW 264.7 macrophages for 24 h with varying concentrations of vitexin (25 μg/mL, 50 μg/mL and 100 μg/mL), the IC_50_ of vitexin was greater than 200 μg/mL, while doxorubicin, used as the positive control, showed IC_50_ of 4.8 ± 2.5 μg/mL, indicating that vitexin did not seem to produce cytotoxicity in in vitro. Vitexin was also observed to be safe with respect to liver damage and gastric mucosa injuries in mice model, as assessed over 7 days of treatment (63). This dependable safety profile of vitexin makes it a potential therapeutic candidate for many diseases, including PD.

### Pharmacological Properties of Vitexin

**Figure 2:**
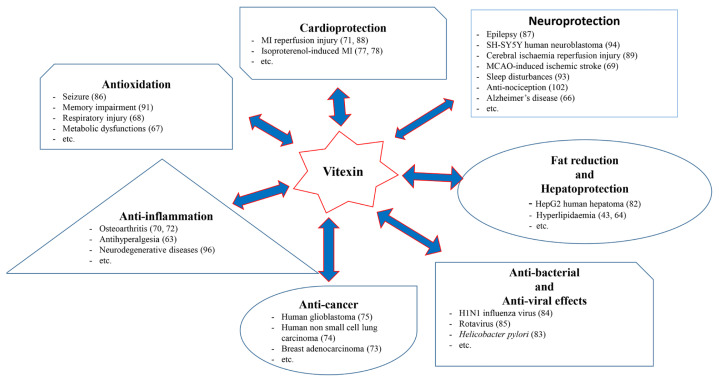
A schematic diagram representing vitexin’s therapeutic potential in some disease conditions. Notes: MI = myocardial injury; MCAO = middle cerebral artery occlusion

In recent years, vitexin has gained considerable momentum as a beneficial and health-promoting agent because of its relatively better safety profile and multi-modal mechanism of action compared to other structurally related flavones (29). A plethora of studies have demonstrated the therapeutic potential of vitexin in several diseases (Figure 2). The suggested therapeutic potential exhibited by vitexin is primarily attributed to its strong antioxidant, anti-inflammatory, neuroprotective, anti-cancer, anti-microbial, cardio-protective, hepato-protective and fat modulatory properties, among others (29).

#### Antioxidant Property of Vitexin

Oxidative stress is a state of disequilibrium between reactive oxygen species (ROS), such as the free radicals, and the biological defence antioxidant systems, resulting in increased cell toxicity and physical damage to cells by lipid peroxidation and alteration of nucleic acids and proteins (64). This is one of the major pathogenic mechanism in virtually all the chronic diseases, such as cancers, metabolic diseases, cardiovascular and neurodegenerative diseases among others (23). Many in vivo and in vitro studies have proven the potent antioxidant properties of vitexin in many oxidative stress-related diseases (65–67). The general antioxidant property of vitexin is mediated by its ability to increase cell viability via intracellular scavenging of ROS and malondialdehyde (MDA) levels (63, 68). It also ameliorates tissue damage by enhancing the activities of antioxidant enzymes, such as superoxide dismutase (SOD), catalase, NAD (P) H: quinone oxidoreductase-1 (NQO-1), heme oxygenase-1 (HO-1), glutathione peroxidase (GPx) and glutathione reductase (GR), among others (64). At the molecular level, it has the potential to upregulate antioxidant response proteins, such as 5-adenosine monophosphate-activated protein kinase (AMPK) and nuclear factor erythroid 2-related factor 2 (Nrf2) (27). In a nutshell, vitexin could be regarded as a robust antioxidant that can be used to prevent diseases induced by oxidative stress.

#### Anti-Inflammation Property of Vitexin

Numerous in vivo and in vitro studies have demonstrated that the anti-inflammatory activity of vitexin can be attributed to its ability to downregulate the pro-inflammatory cytokines, like tumour necrosis factor (TNF)-α, interleukin (IL)-1β and IL-6, and pro-inflammatory enzymes, such as inducible nitric oxide synthase (iNOS), myeloperoxidase (MPO), cyclooxygenase-2 (COX-2) and matrix metalloproteases (MMP) (62, 69, 70). At molecular level studies have also shown that vitexin can inhibit pro-inflammatory mediators release via inhibition of nuclear factor-kB (NF-kB), p38 mitogen activated protein kinase (MAPK), extracellular signal-regulated protein kinase (ERK)1/2 and c-Jun N-terminal kinase (JNK) signalling pathways (62, 69). Furthermore, vitexin also enhances the activities of anti-inflammatory cytokines, such as IL-4 and IL-10 (71, 72).

#### Anti-Cancer Property of Vitexin

The potent antineoplastic effects of vitexin in various types of cancers in organs and systems, such as breast cancer, liver cancer, colorectal cancer, lung and skin cancer, oral cancer, esophageal cancer, ovarian, cervical and prostate cancer and leukaemia has been demonstrated in many in vivo and in vitro studies (73–75). A previous study has demonstrated that the mechanism of protective action of vitexin varies depending on the cancer type (76). In general, vitexin can exert its antineoplastic action by targeting multiple pathways, such as:

- Cell growth inhibition via downregulation of phosphatidyl inositol-3 kinase (PI3K)/Akt, mammalian target of rapamycin (mTOR) and MAPK signalling pathway
- Induction of apoptosis and autophagy through the upregulation of p53, p53-upregulated modulator of apoptosis (PUMA), Bcl2-associated X protein (Bax), poly (ADP-ribose) polymerase (PARP), p-JNK, cytochrome C (CytC), Fas receptor-Fas ligand (Fas/FasL) and MAPK, and downregulation of the caspases, B-cell lymphoma 2 (Bcl-2) and ERK1/2
- Arresting the cell cycle by downregulating Cyclin and cyclin-dependent kinases
- Inhibiting angiogenesis by downregulating hypoxia inducible factor (HIF) and vascular endothelial growth factor (VEGF) pro-angiogenic factors
- Suppressing cancer spread by decreasing MMPs as well as downregulating the oncogenic proteins NF-κB (76)

The potent anti-inflammatory and antioxidant properties of vitexin can also affect multiple signalling pathways relevant to the metastatic growth, proliferation and progression (64, 76).

#### Cardio-Protective Property of Vitexin

Endothelial cell dysfunction, inflammation, oxidative damage and lipid peroxidation are the key pathogenic players in the development of various cardiovascular diseases, including myocardial injury, atherosclerosis, hypertension and cardiac myopathies, among others (29, 64). Some in vivo and in vitro studies have revealed that vitexin can attenuate these pathways, leading to cardiovascular disease development in different cardiovascular disease models (77, 78). Vitexin can mediate its cardio-protective effects by activating the AMPKα signalling pathway, which enhances physical stress resistance and cell viability by decreasing lactate dehydrogenase (LDH) and creatinine kinase (CK) release (79). In cardiac endothelial cell dysfunction, vitexin can upregulate cell autophagy by activating some pro-autophagic genes, such as Beclin-1 and light chain 3 II (LC3-II), and downregulating the p62 anti-autophagic gene (80). Vitexin can also exert it cardio-protective property by inhibiting intracellular free calcium as well as downregulating the calcium downstream effectors, such as calcineurin-nuclear factor of activated T cell 3 (NFATc3) and phosphorylated calmodulin kinase II (CaMKII) (81).

#### Fat Reduction and Hepato-protective Property of Vitexin

Several studies have shown that the fat reduction property of vitexin is mediated by its ability to upregulate the AMPKα signalling pathway for controlling fat accumulation and downregulating CCAT/enhancer binding protein alpha (C/EBPα), Fas and peroxisome proliferator-activated receptor gamma (PPARγ) protein expression levels, which promote lipogenesis and adipocyte differentiation (43, 64). The hepato-protective property of vitexin is demonstrated by its ability to downregulate the liver enzymes, such as alkaline phosphate (ALP), aspartate transaminase (AST), alanine transaminase (ALT) and LDH enzymes (82).

#### Anti-Microbial Property of Vitexin

Vitexin exhibits potent anti-microbial effect especially on a Gram-negative organism, such as *Pseudomonas aeruginosa, Proteus mirabilis, Enterobacter cloacae* and *Escherichia coli*, by exerting anti-biofilm effect primarily via the reduction of a cell adhering ability through inhibition of quorum-sensing regulator proteins and pathogen-swarming motility (29). Quílez et al. (83) demonstrated that the anti-*Helicobacter pyloric* effect of vitexin could be attributed to its ability to inhibit gastric H^+^/K^+^ ATPase, an enzyme that acidifies the stomach. Some studies have also shown that vitexin can inhibit influenza virus neuraminidase, an enzyme responsible for influenza virus replication and release within the host (84, 85).

#### Neuro-Protection Property of Vitexin

Oxidative stress injury and neuroinflammation are the major pathogenic events associated with neuronal loss in virtually all the disorders linked to the neurons, such as seizure and epilepsy (86, 87), retinal damage (88), hypoxic-ischaemic injury (89), depression (90), cognitive dysfunction (91, 92), sleep disorders (93) and neurodegenerative diseases (25, 94), among others. Vitexin can attenuate ROS release in neuron disorders by activating the antioxidant response protein Nrf2, which, in turn, upregulates the activities of antioxidant enzymes (89, 94). It also acts by inhibiting the NF-kB factor, which downregulates the release of pro-inflammatory mediators in neuronal disorders (95). In some neuronal disorders, vitexin can promote neuron survival by activating Nrf2, which increases the expression of brain-derived neurotrophic factor (BDNF) and enhances the antioxidant response (64). Vitexin has also been shown to exert its neuroprotective effect by activating the P13K/AKT signalling pathway, which upregulates the release of antiapoptotic genes/proteins (Bcl 2, BDNF and Nrf2) and downregulates the release of the pro-apoptotic genes/proteins [caspases, Bax and Bclx/Bcl2-associated death promoter (Bad)] (89, 94).

### Possible Molecular Mechanisms Underlying the Neuroprotective Potential of Vitexin in Parkinson’s Disease Pathogenesis

Various mechanisms have been suggested to underlie the neuroprotective actions of vitexin in neurodegenerative diseases, including inhibiting neuroinflammation, attenuating oxidative stress, inhibiting abnormal protein aggregation, downregulation of pro-apoptotic proteins and upregulation of the pro-survival proteins, among others (96). This section shall dwell on the salient pharmacological properties of vitexin that relate to the general pathogenesis of neurodegenerative diseases and extrapolate them to the molecular mechanisms of action in PD pathogenesis.

#### Oxidative Stress in Parkinson’s Disease and the Protective Role of Vitexin

Oxidative stress in one of the major intrinsic players identified in the pathogenesis of PD (97). The high metabolic level coupled with the high levels of reducing iron and polyunsaturated fatty acids present in the dopaminergic neuron of PD patients predisposes the DA neurons to oxidative damage (98). The ROS that induce oxidative damage are mainly produced endogenously from many enzymatic and metabolic events in the body, such as during the activities of inducible nitric oxide synthase (iNOS), endothelial nitric oxide synthase (eNOS), peroxisome oxidases, xanthine oxidases, nicotinamide adenine dinucleotide phosphate oxidase (NADPH oxidases), iron, inflammatory cytokines and cytochrome P-450, among others (97). These reactions promote excessive production of ROS, such as hydrogen peroxide (H_2_O_2_), nitric oxide (NO) and hydroxyl (OH) radicals (97). These ROS overwhelm cellular antioxidant defence mechanism and causes DA nigrostriatal cell damage in SNpc and striatum through lipid peroxidation, DNA damage, inflammation and protein modification (97).

The Nrf2 is a key transcription factor present in all humans and the master regulatory protein against oxidative damage that has been implicated in the pathogenesis of PD (99). In a physiological state, the Nrf2 is found in the cytosol bound to and regulated by Kelchlike erythroid cell-derived protein with CNC homology (ECH)-associated protein 1 (Keap1) (99). However, is a stressful state, Nrf2 passively dissociates from Keap1; then, DJ-1 protein stabilises it and the Nrf2 is finally translocated into the nucleus. This nuclear translocation of Nrf2 is regulated via phosphorylation by ERK1/2 and PI3k/Akt (97). The nuclear Nrf2 interacts with antioxidant response element (ARE) in the promoter region of the cytoprotective genes and upregulates the expressions of antioxidants enzymes, like SOD, catalase, HO-1, GPx, NQO-1 and GR among others (99). It eventually ameliorates tissue damage.

**Figure 3:**
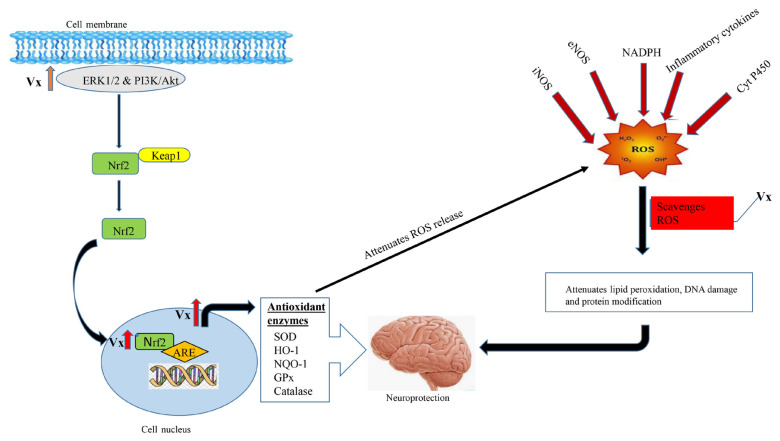
Signalling pathway mediating vitexin antioxidant properties in PD. Vitexin enhances the expressions of ERK1/2 and PI3k/Akt, which, in turn, increase Nrf2 concentration. Nrf2 upregulates antioxidant enzymes that can attenuate tissue damage and ROS release, and hence, provides neuroprotection Notes: ARE = antioxidant response element; Cyt P450 = cytochrome P450; ERK = extracellular signal-regulated protein kinase; eNOS = endothelial nitric oxide synthase; GPx = glutathione peroxidase; HO-1 = heme oxygenase-1; iNOS = inducible nitric oxide; Keap1 = Kelch-like erythroid cell-derived protein with CNC homology (ECH)-associated protein 1; Nrf2 = nuclear factor erythroid 2 related factors; NQO-1 = Quinone oxidoreductase-1; NADPH = Nicotinamide adenine dinucleotide phosphate oxidase; PI3K/Akt = phosphatidyl inositol-3 kinase/Akt; ROS = reactive oxygen species; SOD = superoxide dismutase; Vx = vitexin

Pre-clinical studies have demonstrated that natural flavonoids, such as vitexin, may confer substantial antioxidant effect against oxidative damage, which is one of the basic pathogenic mechanisms detected in neurodegenerative diseases, including PD (96). In a study conducted by Aseervatham et al. (87), the neuroprotective effect of vitexin in pilocarpine-induced epileptic mice was achieved by its ability to freely quench ROS and upregulate antioxidant enzymes (SOD, catalase and GSH). In a human brain microvascular endothelial cells (HBMEc) ischaemia/reperfusion injury model, vitexin maintained blood-brain barrier integrity and increased tight junctions proteins expression by freely scavenging intracellular nitric oxide (NO) and perioxynitrite radical (ONOO-) and dampening eNOS and iNOS activities by upregulating antioxidant enzymes (89). In another study by Jiang et al. (69), vitexin was observed to attenuate the deleterious effect of ROS, LDH, malondialdehyde (MDA) and NO in a middle cerebral artery (MCAO)-induced cerebral ischaemic stroke via the mTOR/U1k1 pathway. The pre-treatment of Neuro-2a cells with vitexin inhibited oxidative stress-mediated damage in glutamate toxicity manifested in Alzheimer’s disease by augmenting the expression of antioxidant response genes pathway (Nrf-2/HO-1 and NQO-1) (66). Vitexin was also proven to protect PC12 cells against hypoxia/re-oxygenation-induced injury by suppression of NADPH oxidase and subsequent reduction of ROS production (100). In another study by Zhang et al. (79), vitexin reduced ox-LDL-mediated endothelial injury by inhibiting the production of ROS and MDA and also by promoting the expression of SOD. In another study, vitexin was observed to improve spatial learning and memory in diabetic rats via upregulation of activities of antioxidant enzymes SOD and GPx (92). Based on these preclinical shreds of evidence, it is also speculated that this antioxidant property of vitexin can combat the oxidative stress manifested during the pathogenesis of PD. Vitexin can freely scavenge ROS in PD pathogenesis, thereby conferring neuroprotection (Figure 3). It can also act by upregulating the ERK1/1 and PI3K/Akt signal pathway, which enhances nuclear translocation of Nrf2, and thus, increases antioxidant enzymes production, which helps in ameliorating neuronal tissue damage (Figure 3). Rahman and Kumar (59) found that vitexin-loaded lipid nanoparticles-based therapeutics in 6-OHDA induced PD mice model improved the levels of total reactive antioxidant enzymes, including CAT, SOD, glutathione (GSH), GPx and glutathione s-transferase (GST), thereby providing neuroprotection.

#### Neuro-Inflammation in Parkinson’s Disease and the Protective Role of Vitexin

**Figure 4:**
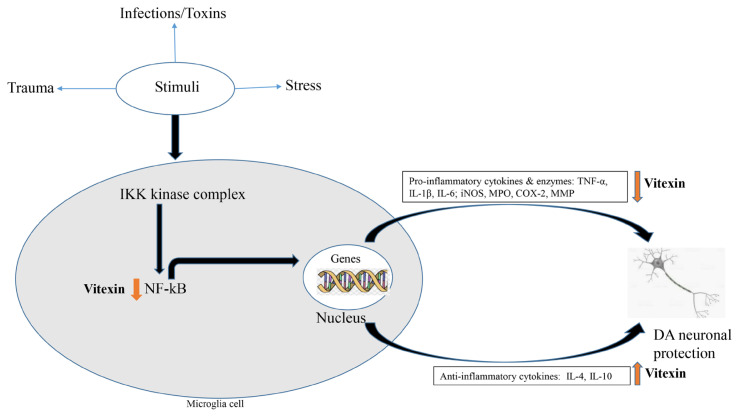
Signalling pathways mediating vitexin-mediated anti-inflammatory properties in PD Notes: COX-2 = cyclooxygenase-2; DA = dopaminergic; IL = interleukin; iNOS = inducible nitric oxide synthase; MPO = myeloperoxidase; MMP = matrix metalloprotease; NF-kB = nuclear factor-kB; TNF-α = tumour necrosis factor-alpha

Neuroinflammatory processes also play a critical role in the development of PD (12). Pro-inflammatory signals, such as infection, trauma, stress or exposure to environmental factors can directly activate microglial cells. Activated microglial cell via its ‘master switch’ regulatory transcription factor nuclear factor-kB (NF-kB) enhances the synthesis and release of the pro-inflammatory cytokines, such as TNF-α, IL-1β and IL-6, and pro-inflammatory enzymes, such as iNOS, MPO, COX-2 and MMP, which might contribute in apoptotic cell death of dopaminergic neurons (101). A lot of in vivo and in vitro studies have demonstrated that vitexin can directly inhibit the NF-kB transcription factor, and thus, downregulate the production of pro-inflammatory cytokines/enzymes (62) and upregulate the anti-inflammatory cytokines (IL-4 and IL-10) (63, 102). It is also tempting to assume that vitexin can inhibit NF-kB transcription factor in PD pathogenesis, which, in turn, downregulates pro-inflammatory mediators and upregulates the anti-inflammatory mediators, and thereby, confers DA neuronal protection (Figure 4). Although, to the best of our knowledge, there are no documented studies in PD models to support this speculation, we strongly believe vitexin to be a promising candidate to protect against inflammation during PD pathogenesis.

#### Modulatory Role of Vitexin in Apoptosis and Cell Survival in Parkinson’s Disease

**Figure 5:**
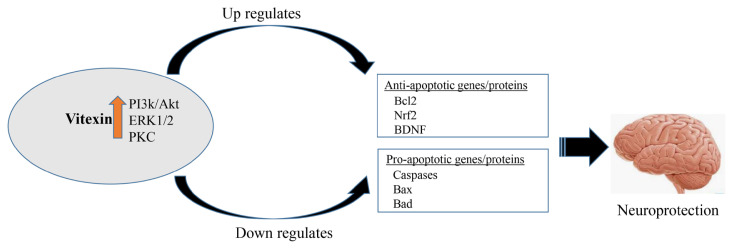
Pathway showing the effects of vitexin on cell survival and apoptosis in PD Notes: Bcl2 = B-cell lymphoma 2; Bax = Bcl2-associated X protein; Bad = Bclx/Bcl2-associated death promoter; BDNF = brain-derived neurotrophic factor; ERK = extracellular signal-regulated protein kinase; Nrf2 = nuclear factor erythroid 2 related factors; PI3K/Akt = phosphatidyl inositol-3 kinase/Akt; PKC = protein kinase C

Several flavonoids, including vitexin, can interact with some important cell survival signalling pathways, such as PI3k/Akt, ERK1/2 and protein kinase C (PKC), and protect against neurodegeneration in PD (12). The activation of these pathways imparts beneficial effects on cell survival via upregulation of anti-apoptotic genes, such as Bcl2, Nrf2 and BDNF and inhibition of pro-apoptotic proteins (caspase 3 and 9, Bax and Bad, among others) (Figure 5) (12). A recent study demonstrated that vitexin protected DA neurons against MPP+/MPTP-induced neurotoxicity by upregulating the PI3K/Akt signalling pathway, which suppressed the ratio of Bax/Bcl2 and caspase-3 activities (94). Although we found only one study of this kind that investigated the neuroprotective role of vitexin, we believe that, combined with the results of other studies that demonstrated pro-cell survival property of flavonoids in neurodegenerative diseases, it can be hypothesised that vitexin can potentially be used as a potent therapeutic agent against PD. However, more studies are still warranted in this domain.

#### Protein Mis-folding and Aggregation in Parkinson’s Disease and the Protective Role of Vitexin

**Figure 6:**
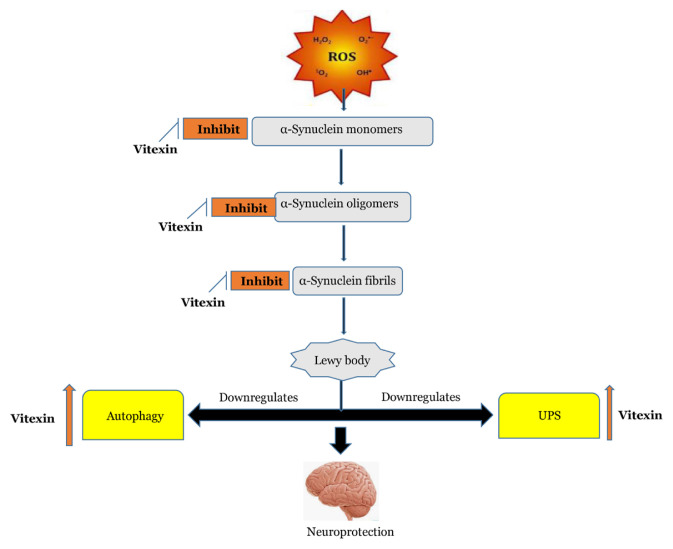
Pathway mediating the effects of vitexin on protein mis-folding and aggregation in PD Notes: (↑) = upregulates; ROS = reactive oxygen species; UPS = Ubiquitin proteasome system

Increase in ROS causes the production of alpha-synuclein monomers (97) (refer Figure 6). As the level of these monomers increases, they aggregate together to form toxic alpha-synuclein oligomers. Oligomers of this kind can also be produced by mutation of the alpha-synuclein gene (103). The oligomers inhibit ubiquitin-proteasome system (UPS) and autophagy system (ATGS), which are responsible for maintaining biochemical balance in the neurons (104). Failure of UPS and ATGS leads to the development of Lewy bodies, which are one of the pathological hallmarks of PD (105).

Several flavonoids, such as quercetin, curcumin, apigenin, naringenin, epigallocatechin and chrysin, among others, have been shown to possess inhibitory properties against protein mis-folding and aggregation in neurodegenerative diseases, including PD (1, 12, 105). Given the known action of some flavonoids as protein mis-folding and aggregation antagonists, it is also tempting to speculate that vitexin could also possess this beneficial property of inhibiting α-synuclein oligomerisation, fibrillisation and Lewy body formation, and could also upregulate UPS and ATGS, which thus confers neuroprotection in PD (Figure 6). More research is still warranted in this direction.

#### Protective Role of Vitexin in Dopamine Metabolism in Parkinson’s Disease

A previous study has demonstrated that vitexin treatment may increase striatal dopamine content by acting as an inhibitor of monoamine oxidase B (MAO-B) enzyme in 6-hydroxy dopamine (OHDA)-induced mice model (59). In this scenario, vitexin dampened dopamine metabolism and subsequently increased the striatal dopamine levels. It was also reported that it increased the striatal levels of dopamine metabolites, such as homovanilic and 3, 4-dihydroxyphenyl acetic acid (59). These changes were accompanied by the enhancement of memory via a Morris water maze test as well as depression-like behaviour in tail suspension test (59). More studies are encouraged in this direction, as vitexin may write a good end in the story of PD.

## Conclusion

A limited number of studies regarding the neuroprotective potential of vitexin in PD patients have been done. In the same vein, there is no clinical evidence regarding the protective role of vitexin in PD patients; however, it is speculated to impart immense clinical benefits. The promising pharmacological properties of vitexin, including antioxidant and anti-inflammatory activities, the inhibitory effects on protein misfolding and aggregation, procell survival effects and its ability to act as an inhibitor of MAO-B enzyme, thereby increasing the dopamine levels in the brain, makes it stand out amid other flavonoids. Vitexin could be a game-changer in the novel therapeutic strategies of PD. However, more pre-clinical and clinical studies are needed to provide a robust argument for the possible neuroprotective potential of vitexin in PD patients, and hence, its clinical application.
